# Evidence-Based interventions of Norovirus outbreaks in China

**DOI:** 10.1186/s12889-016-3716-3

**Published:** 2016-10-12

**Authors:** Tianmu Chen, Haogao Gu, Ross Ka-Kit Leung, Ruchun Liu, Qiuping Chen, Ying Wu, Yaman Li

**Affiliations:** 1 Office for Disease Control and Emergency Response, Changsha Center for Disease Control and Prevention, 149 Wei’er Road, Changsha, Hunan People’s Republic of China; 2 School of Public Health, The University of Hong Kong, Hong Kong SAR, People’s Republic of China; 3 Stanley Ho Centre for Emerging Infectious Diseases, The Chinese University of Hong Kong, Hong Kong SAR, People’s Republic of China; 4 Hospital, Shanghai Normal University, Shanghai, People’s Republic of China; 5 Office for Disease Control and Emergency Response, Tianxin Center for Disease Control and Prevention, Changsha, Hunan People’s Republic of China

**Keywords:** Mathematical model, Norovirus, Outbreak, Water disinfection

## Abstract

**Background:**

In resource-limited settings where laboratory capacity is limited and response strategy is non-specific, delayed or inappropriate intervention against outbreaks of Norovirus (NoV) are common. Here we report interventions of two norovirus outbreaks, which highlight the importance of evidence-based modeling and assessment to identify infection sources and formulate effective response strategies.

**Methods:**

Spatiotemporal scanning, mathematical and random walk modeling predicted the modes of transmission in the two incidents, which were supported by laboratory results and intervention outcomes.

**Results:**

Simulation results indicated that contaminated water was 14 to 500 fold more infectious than infected individuals. Asymptomatic individuals were not effective transmitters. School closure for up to a week still could not contain the outbreak unless the duration was extended to 10 or more days. The total attack rates (TARs) for waterborne NoV outbreaks reported in China (*n* = 3, median = 4.37) were significantly (*p* < 0.05) lower than worldwide (*n* = 14, median = 41.34). The low TARs are likely due to the high number of the affected population.

**Conclusions:**

We found that school closure alone could not contain Norovirus outbreaks. Overlooked personal hygiene may serve as a hotbed for infectious disease transmission. Our results reveal that evidence-based investigations can facilitate timely interventions of Norovirus transmission.

**Electronic supplementary material:**

The online version of this article (doi:10.1186/s12889-016-3716-3) contains supplementary material, which is available to authorized users.

## Background

Neither specific treatment nor vaccination is available to norovirus illness. Despite a highly contagious virus that causes gastroenteritis worldwide, the monitoring of NoV is elusive. In the United States, NoV was responsible for annual incidence of 21 million [1], 71,000 hospitalizations [2] and 800 deaths [1, 3]. In developing countries, NoV outbreak reports are limited and NoV was found to be responsible for diarrheal disease in The Gambia [4]. The paradoxical higher prevalence of NoV in controls than in cases [4] was suggested as a result of reinfection by mathematical modeling [5]. Although the capacity of norovirus nucleic acid detection is becoming available in China, the understandings towards this pathogen remain accumulating, more formal reports of NoV outbreaks are needed [6–8]. Waterborne NoV outbreaks were reported worldwide and some of the reported outbreaks since 2000 are summarized in Additional file 1: Table S1. Most of the outbreaks were related to potable water contamination. Dubbed winter vomiting bug, waterborne NoV outbreaks indeed appeared to occur all year round.

Even for developed countries, mathematical modeling studies of NoV are limited. Neither estimates of the basic reproduction number *R*_0_ nor other relevant parameters were estimated for developing countries [5]. An estimate of *R*_0_ = 3.74 was obtained by fitting a compartmental transmission model to a NoV outbreak data involving 39 individuals in Belgium [9].

An uncontained outbreak could have severe economic consequences [10]. Ward closure to control nosocomial NoV outbreak was also not advocated, concerning bed-days and revenue loss [11–13]. Campus NoV outbreak was also reported in California, Michigan and Wisconsin in 2008, widespread infection and rapid transmission prompted closure of the Michigan campus [14]. School closure is a common method to contain NoV transmission in China. However, the effectiveness is unknown. Furthermore, despite China's improvement in water, sanitation and hygiene, more than 320 million people still lacked access to sanitized water [15]. The vulnerability is to be explored.

Although the factors of incubation period, latent period, infectious period, the proportion of latent infection for NoV were investigated [16–25], the corresponding factors of NoV transmission in the community, schools and via water and the contribution of asymptomatic individuals to the transmission have not been elucidated. In China, there is a lack of quantitative assessment of the effectiveness of interventions such as isolation, water disinfection and school closure. The aforementioned factors and effectiveness of countermeasures are difficult to characterize by conventional epidemiological methods. Mathematical modeling and simulation can complement the analysis [26, 27]. Here we report two outbreaks in a China metropolis, Changsha City, and compared the outbreak reports with those of the worldwide. Basing on the natural history of diarrhea due to NoV infection, we built an ordinary differential equation (ODE) model to characterize the NoV interpersonal and waterborne transmission dynamics and the effectiveness of intervention in schools and communities.

## Methods

The study and investigation were in response to an acute public health emergency event, and no personal identifiable information was include. Ten to fifty percent of the total population at risk were used for space-time scanning by SaTScan™ v9.4.2 (July 2015, Boston and Information Management Services Inc., Calverton Maryland). The significance of mode of transmission was estimated by permutation tests on the basis of Monte Carlo simulations [28]. Random walk was used to sample the probability distribution of interpersonal transmission [29]. Two states “interpersonal” and “non-interpersonal” were modeled. In the first incident, random walk modeling was used to assess the proportion between interpersonal and waterborne transmissions. In the second incident, the visit frequency to the potential source of infection was estimated.

A Susceptible-Exposed-Infectious/asymptomatic-Removed-Water (SEIARW) model, similar to the one modeling shigellosis outbreak [26], was used to characterize NoV transmission epidemics. Berkeley Madonna 8.3.18 (developed by Robert Macey and George Oster of the University of California at Berkeley. Copyright ©1993-2001 Robert I. Macey & George F. Oster) was employed for model simulation. The Runge-Kutta method of order 4 with the tolerance set at 0.001 was used to perform curve fitting on prevalence [30]. Sensitivity analysis was tested by varying parameters into 1000 values ranging from the minimum to maximum of the reported values in literature (Additional file 2: Table S2).

## Results

### Incident 1

On December 7, 2014, continual vomiting and diarrhea cases were reported in different locations of a village in Changsha. For the field outbreak investigation, some case definitions were defined as follows: a), probable case was defined as vomiting or diarrhea (>3 times/day) in the village population since November 25, 2014, additional symptoms included nausea, fever, chill, abdominal pain, abdominal distension, headache and dizziness; b) confirmed case was defined as probable case plus positive of Norovirus from the feces or vomitus samples; c) asymptomatic was defined as positive fo Norovirus but without any symptoms (in this outbreak, asymptomatic infection was not investigated by local CDC but simulated by the SEIARW model). Some residents in the affected community had manifested symptoms such as vomiting or diarrhea (>3 times/day), fever, headache and dizziness since November 28. Since the index case dated November 28, the number of cases increased and remained high with fluctuation from December 3 until it flatten off on December 7. Demographic and clinical characteristics of the cases manifested symptoms of gastroenteritis (Additional file 3: Table S3). Preliminary epidemiologic field investigations identified the village had its own deep wells (depth 280 m) and no other water sources were nearby. Three pumping stations delivered water to the neighborhood for drinking and household use but the extracted groundwater was not disinfected. In spite of sampling from various sources such as rectal swabs, water from different locations, both laboratory test and field investigation took time and therefore isolation was implemented first.

The period between November 28 and December 7 was regarded as a non-intervention period. The SEIARW model was used to estimate the interpersonal transmissibility *b*, the water-to-person transmissibility *b*_*W*_, the transmissibility ratio of asymptomatic-to-symptomatic individuals *k*, the asymptomatic individual viral shedding coefficient *c,* and the basic reproduction number *R*_0_. There were 643 residents in the village. At the beginning there was one case, therefore s_0_ = 0.998, e_0_ = 0, i_0_ = 0.002, a_0_ = 0, r_0_ = 0, w_0_ = 0 (Please see Additional file 4 for more details) respectively. If there is excreted virus within water, then the water body becomes infectious. Since w(t) is a constant, sensitivity test showed that w(t) = 1 is the best fit. The following parameters were estimated accordingly: *b* = 0.8230, *b*_*W*_ =0.01470, *k* = 4.1539 × 10^-11^, *c* =0.182201, *R*_0_ = 1.94 and *R*_0W_ = 4.91 (Additional file 5: Figure S1). Therefore, asymptomatic individuals were not prioritized as the targets of analysis and infection control. Infection control team expanded the field investigation in order to evaluate the sources of infection on the 8^th^ December.

**Fig. 1:**
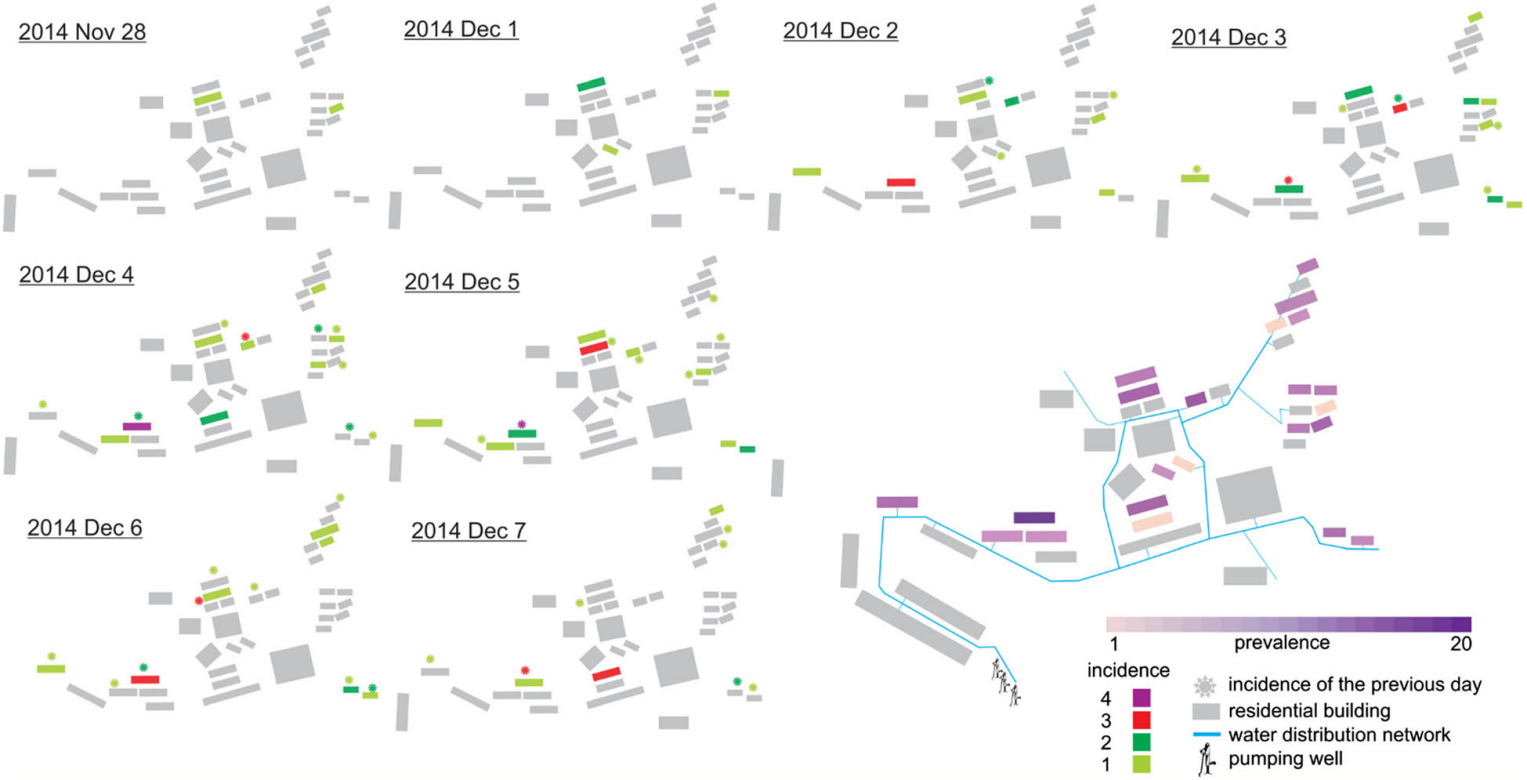
Spatiotemporal information of reported cases and the water distribution network of the village outbreak

The team focused on water contamination and drew a map showing the spatiotemporal information of 90 reported cases and the water distribution network (Fig. 1). Space-time permutation scan did not reveal disease clusters. Integrating the information available from the depth of well (280 m) and the spatiotemporal distribution of the cases, design and maintenance of water service and building sewer, contamination of water pipes but not the well or pumping stations was likely. Field epidemiological survey showed that water pipes were all iron and were used more than decades of years, and some of which were buried under land, some of them were closed to surface drain or sewage pool, which might increase the probability of the contamination although local CDC did not find the contaminated spot. We could strongly infer that some spot of the pipe was rusty and contaminated, and then the contaminated water would go through widely when the pump worked and make a suck-back. More information about the water distribution network and storage devices was included in the Additional file 6.

**Fig. 2:**
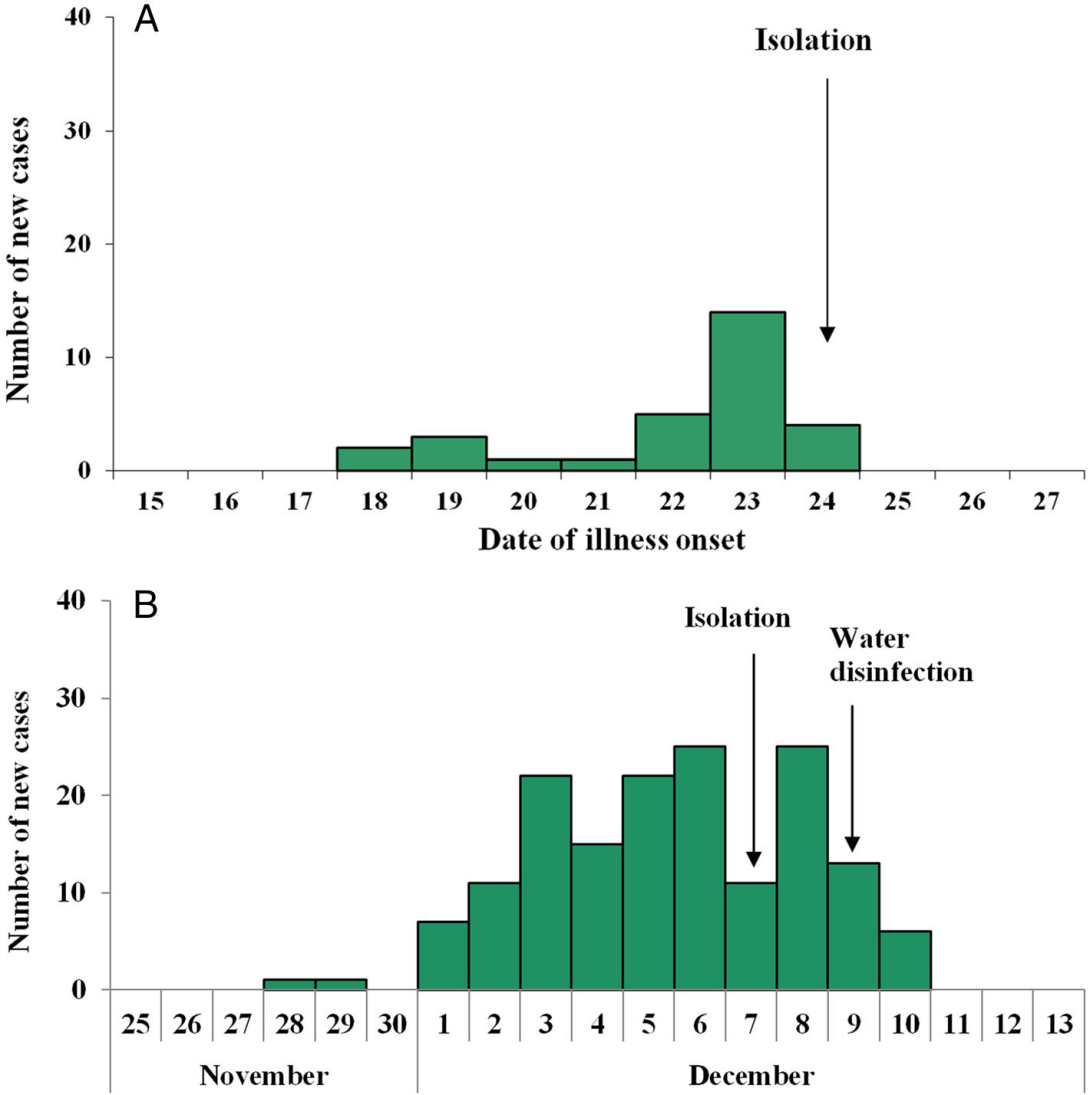
Distribution of cases by date of illness onset. **a** the school outbreak. **b** the village outbreak

On the 9^th^ of December laboratory results were available. Rectal swab results were negative for *Vibrio cholerae* O1 or O139, *Vibrio cholerae, Escherichia coli, Proteusbacillus vulgaris, Salmonella* and *Shigella* species and other common diarrheal pathogens (Rotavirus, Adenovirus, Astrovirus and Sappovirus), but positive for NoV GII. Total coliforms, thermotolerant coliforms, and colony count for bacteria all exceeded critical values in tap water samples. NoV GII was again tested positive. Solid form of chlorine was then put into water towers of pumping stations. The epidemic began to decline after disinfection and on the 13^th^ of December no more new cases were identified. The epidemic curve (Fig. 2) indicates a pattern of mixed epidemic. There were a total of 159 cases, affecting about 643 residents. The total attack rate was 24.73 % and the outbreak lasted for 13 days.

**Fig. 3:**
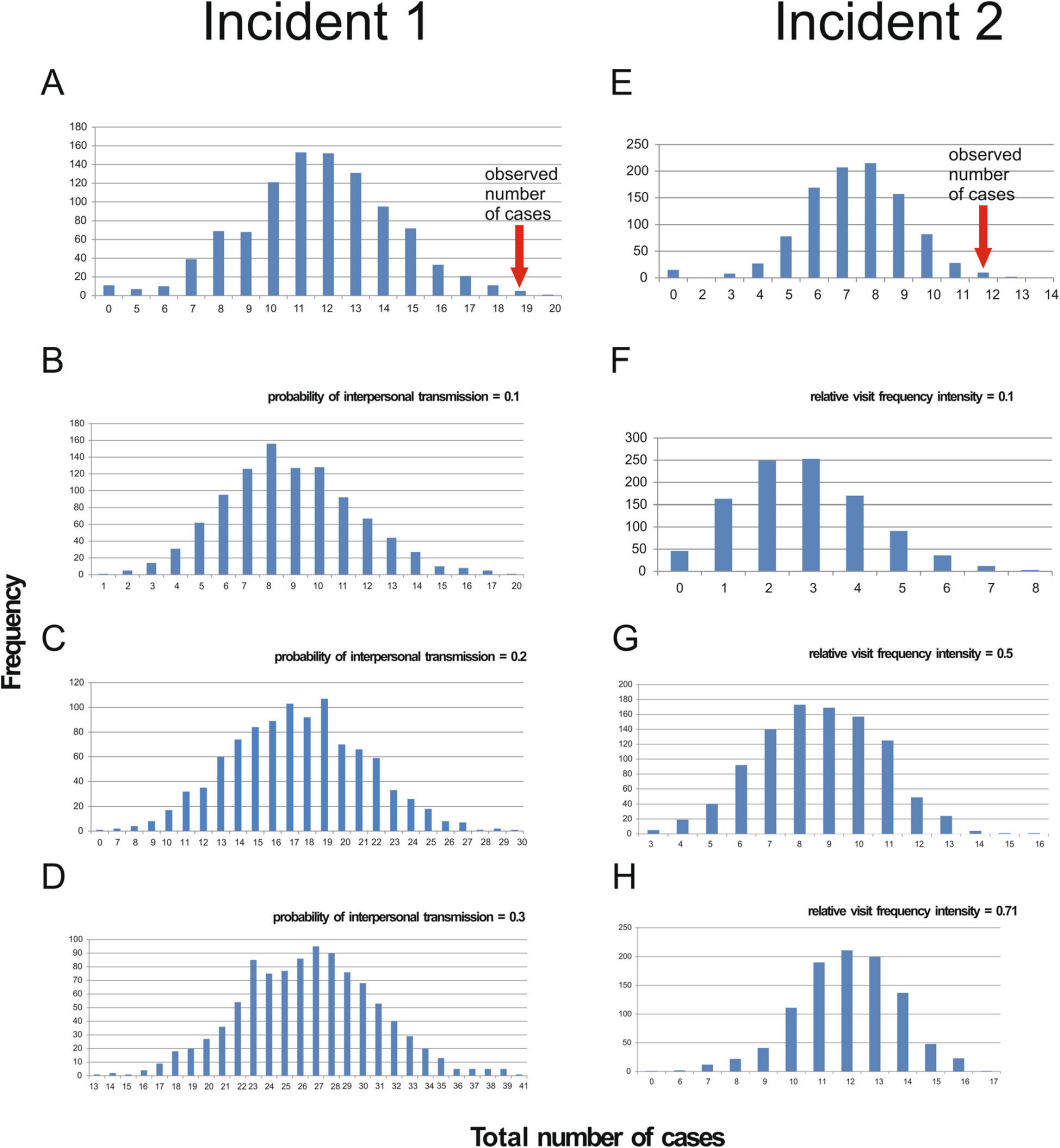
Permutation test results by random walk modeling for incidents 1 (**a**-**d**) and 2 (**e**-**h**). **a** The observed number of 19 cases in incident 1 was unlikely to be due to pure waterborne infections. **b**-**d** Simulated number of cases by different probability of interpersonal transmission from 1000 experiments. **e** The observed number of 12 cases in incident 2 was unlikely to be due to random infections. **f**-**h** Simulated number of cases by different relative visit frequency intensity from 1000 experiments

The isolation measures taken during December 7-8 had not alleviated the epidemic. We therefore conducted a retrospective analysis of SEIARW modeling, employing the full-course data of the outbreak to assess the effectiveness of isolation, water disinfection and the combined approach. Simulation results showed that isolation alone still results in a TAR of 69.99 % (95 % CI, 69.34-70.64), comparable to the situation of no intervention. Water disinfection isolation alone did better, but still unable to contain the outbreak (TAR dropped to 53.89 % (95 % CI, 53.32-54.46)). The TAR and the duration of outbreak (DO) of combined measure were most consistent with the observed data (25.32 vs 24.73 and 23 vs 23, respectively). More details of the simulation results are shown in Table 1. Among 22 affected apartments, there were 19 flats each with two reported cases happening on the same or next day. Monte Carlo permutation test result (Fig. 3a) suggests that this was unlikely to happen by chance (*p* = 0.01), which supports the contribution of interpersonal in addition to water-to-person transmission. Random walk results suggest that interpersonal transmission contributed to about 20 percent of all cases (Fig. 3b-d).

**Table 1:** Comparison between reported data and simulated results by different interventions implemented in two outbreaks in Changsha, 2014

Intervention	TAR^a^ (%)		Cumulative cases	DO^c^(day)
	%	95 % CI^b^		
Outbreak 1				
Reported data	24.73	24.35-25.11	159	23
Isolation	69.99	69.34-70.64	450	190
Water disinfection	53.89	53.32-54.46	346	43
Isolation + Water disinfection	24.93	24.54-25.32	160	23
None	70.00	69.35-70.65	450	103
Outbreak 2				
Reported data	2.14	2.06-2.22	30	16
Isolation	2.26	2.18-2.34	32	15
School closure (7 days)	67.23	66.80-67.66	941	50
School closure (8 days)	67.22	66.79-67.65	941	52
School closure (9 days)	67.21	66.78-67.64	941	54
School closure (10 days)	2.26	2.18-2.34	32	15
Isolation + School closure (7 days)	2.26	2.18-2.34	32	15
Isolation + School closure (8 days)	2.26	2.18-2.34	32	15
Isolation + School closure (9 days)	2.26	2.18-2.34	32	15
Isolation + School closure (10 days)	2.26	2.18-2.34	32	15
None	67.45	67.02-67.88	944	39

^a^Total attack rate; ^b^confidence interval; ^c^duration of outbreak

Simulated results of the best execution time of different interventions revealed that the earlier the combined intervention were simulated the better the effectiveness of the strategy would concluded. Although isolation implemented at different execution time had the same TAR, the earlier the intervention was conducted the lower number of peak cases and the longer DO of the outbreak. The similar results were observed on water disinfection, what the difference was that the TAR was low down to 51.18 %. However, when isolation and water disinfection were combined, the earlier the interventions were conducted the lower TAR and number of peak cases, the shorter DO of the outbreak and the earlier the peak day (Additional file 7: Table S4).

### Incident 2

**Fig. 4:**
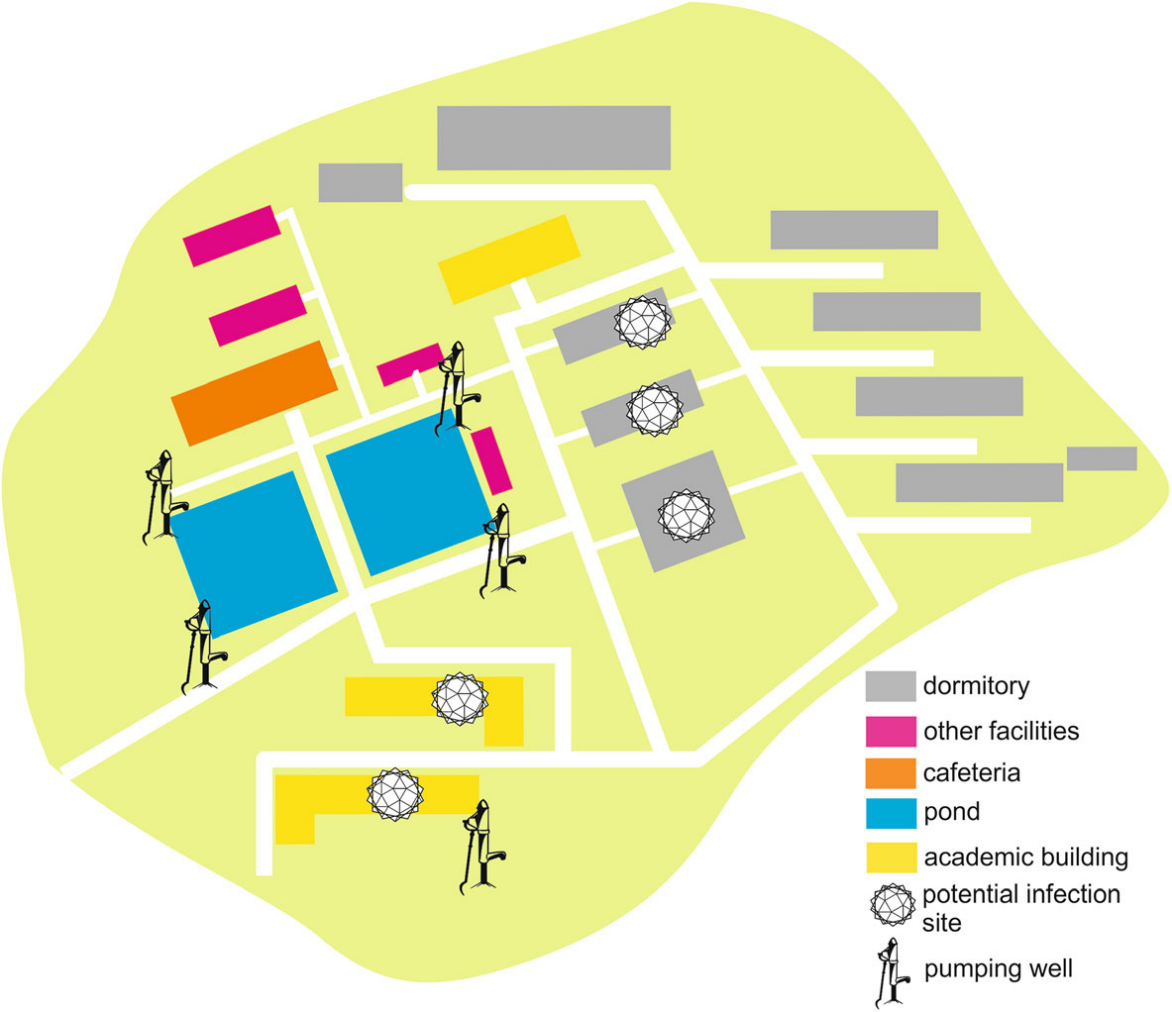
Probable locations of infection of the school outbreak

On December 24, 2014, a local branch Center for Disease Control and Prevention in Changsha received a telephone report about frequent cases of vomiting, diarrhea in a high school. The school had 25 classes of three grades and a total of 1,400 students and 153 teaching and supporting staff. Our infection control team underwent field epidemiological investigation according to the same case definition as incident 1. the school had its five deep wells each about 50 meters deep, with pressure pumps delivering water to school buildings, dormitories and canteen for domestic water use. Water for drinking was first purified before delivering to drinking bottles. There was a single canteen, which serves all students and staff (Fig. 4). Water samples from each well and taps of teaching complex and anal swabs of canteen staff were collected and subjected to laboratory testing. Negative results were obtained. The vomit and stool specimens from only one case were detected with the presence of GII NoV. Along with these lines of evidence and no staff was infected, neither food poisoning nor water transmission was likely. Monte Carlo permutation result also supports interpersonal transmission (Fig. 3e). Believing interpersonal transmission as the main route, we enforced isolation, supplemented by environmental cleaning and disinfection, education on hand hygiene and avoidance of drinking unboiled water, establishment of daily screening and sickness absence recording system. Indeed after intervention there were no new cases. There were a total of 30 cases, leading to a TAR of 2.14 % and DO of 16 days. The epidemic curve indicates a growth pattern (Fig. 2). Results of model fitting reveals that *b* = 1.6344, *k* = 3.7301 × 10^-9^, *R*_0_ = 3.44. Mathematical modeling was performed to assess the effect of control measures. Isolation and school closure are two common interventions of intramural interpersonal transmission in China. Either isolation or 10-day class suspension (or both) could bring down the TAR and DO to 2.26 % and 15 days respectively. School closure for 7, 8 and 9 days since December 24 were not predicted to be able to contain an outbreak (Please see Additional file 8 for more details), yielding a similar result to that of no intervention (Table 1), with TAR over 67 % and DO more than 39 days. Only 11 out of 25 classes were affected in this incident, and the cases appeared to be sporadic (Additional file 9: Table S5). Since no definitive source was identified, we used random walk to assess the visit frequency of the potential source of infection. We modeled the visit frequency within a specific period as a key factor to get contacts with a contaminated source, assuming that the probability of being infected could be relevant to the visit frequency. The simulation results did suggest that the source had to be a rather frequently visited location in order to regenerate the observed epidemic (Fig. 3f-h).

Until now, we have introduced two reported waterborne NoV outbreaks in China and we compared them with other 14 outbreaks worldwide in terms of the actual TARs (Additional file 1: Table S1). The median TAR in China was 4.37, compared with 41.34 worldwide. The difference was significant (Mann-Whitney test, *p* < 0.05). There was however no significant difference between China and the other countries in terms of the duration between the time of onset of the index case and the time when the outbreak was reported (*p* = 0.92).

Simulated results revealed that the earlier isolation was simulated the better the effectiveness of the strategy would be concluded. However, school closure would not have the good results even when it was implemented earlier to day 3 (Additional file 7: Table S4).

## Discussions

To our knowledge, this is the first study to characterize the transmission dynamics and control of a waterborne NoV outbreak. Our data also enhances our understanding towards the NoV outbreak situation in China.

Inefficient transmission of asymptomatic individuals reconciles the finding of the paradoxical higher prevalence of NoV in controls than in cases of the recent Global Enteric Multicenter Study [4]. Our data also enrich the dataset of waterborne NoV outbreaks in China. School closure was not predicted to be an effective control. Identification of target affected population is as important as locating infection source and mode of transmission in order to take prompt countermeasures. In resource-limited setting, a balance between hazards and benefits calls for action to enforce sanitation and water disinfection; and promote personal hygiene to proactively prevent and contain NoV and other waterborne disease outbreaks.

Contaminated water was 14- to 500-fold more infectious than transmission associated with person-to-person contact. Usually water is far less effective in the transmission of NoV. Despite a much lower transmissibility by water (*b*_*W*_ =0.01) than by the infected in a local community (*b* = 0.82) or a school (*b* = 1.63), the spread of NoV by water was still the most rapid (*R*_0_ = 4.91), versus *R*_0_ = 1.94 in community and *R*_0_ = 3.44 in school. A previous mathematical modeling study of NoV suggested that asymptomatic prevalence could be varying [5], however, our results predicted that contribution of asymptomatic individuals to the spread of NoV was minimal, with low interpersonal transmissibility coefficients *k* = 3.73 × 10^-9^ and *k* = 4.15 × 10^-11^ under the school and the community settings respectively. Their shedding capability was also only about 18 % of the symptomatic individuals. Latent infection devoid of gastroenteritis symptoms such as vomiting and/or watery diarrhea may have significantly lowered the transmissibility. Even if they do shed NoV, the titer is lower [23–25]. Therefore, our results suggest a possible alternative explanation for the paradoxical higher prevalence of NoV in controls than in cases of the recent Global Enteric Multicenter Study [4].

Water-to-person transmission was predicted to be the fastest, followed by interpersonal transmission in school, and then interpersonal transmission in the community. Wide coverage of water supply, daily contact and low infectious dose [31] render water an effective vector for NoV infection. In turn, population density and contact frequency are both higher in schools than in community, which accounts for higher interpersonal transmissibility in the former.

In the NoV outbreak due to drinking water source contamination, isolation alone did not contain the outbreak; TAR was predicted to be about the same, but with a longer DO. Similarly, water disinfection alone could only reduce TAR and DO to about 23 % and 58 % respectively. Only a combined measure of both could bring down the TAR and DO from 70.00 % (no intervention) to 24.93 % and 154 (no intervention) to 23 days.

The actual NoV outbreak TARs reported in China was significantly lower than worldwide. Two reasons may be responsible for this finding. Firstly, the number of the affected population in China was reported significantly larger than that worldwide (3915 versus 1845, *P* < 0.05) while the number of infected population was not (294 versus 158, *P* = 0.96). If reported target populations were non-specific, the reported TARs were indeed deflated. Although the Chinese population is large and reports in India and Russia were unavailable, the number of infected was not correspondingly high. Secondly, many worldwide reports of large TAR were specific incidences in a focused area such as excursion or leisure activities in resorts, hotels or small towns. High cost of viral monitoring outbreaks and control [32], insufficient public perception and awareness of NoV, health-seeking motivation and practices of gastroenteritis, immature information-sharing, data collection and dissemination may hinder reports from local schools, departments or other entities. Experience accumulation, regular health structures and expert epidemiologic support at local level are the first steps towards a well-designed sentinel system for health promotion and disease transmission blockage. In the second incident of NoV outbreak, no positive results were obtained from water samples and those collected from food processing staff. Not a single one of the staff indeed manifested symptoms of NoV infection. The relatively high visit frequency obtained by random walk analysis suggests that toilet might act as the hotbed for transmission. In certain areas of China, hand washing after toilet visits before eating or preparing food is not deemed necessary. Moreover, vomitus and stool in the toilet may not be discarded properly due to the prevalence of dry toilets [33]. In future, flushing and keeping the surrounding area of toilets clean should be advocated. Personal hygiene should also be emphasized.

A limitation of this study was that we analyzed data of only two outbreaks, and that five parameters had to be taken from the literature. Nevertheless, our mathematical models reflect well the actual epidemic. Moreover, sensitivity analysis confirms that our model was relatively insensitive to the change in parameter. Finally, we also characterized the transmissibility and viral shedding coefficient during the asymptomatic phase of the disease. Further field test and pathological investigation are warranted for infection control of NoV.

## Conclusions

We found that school closure alone could not contain Norovirus outbreaks. Overlooked personal hygiene may serve as a hotbed for infectious disease transmission. Our results reveal that evidence-based investigations can facilitate timely interventions of Norovirus transmission.

## Additional files

Additional file 1: Table S1. Summary of some of the reported waterborne NoV outbreaks since 2000. (DOC 97 kb)

Additional file 2: Table S2. Parameter definitions and values for sensitivity analysis. (DOC 89 kb)

Additional file 3: Table S3. Demographic and clinical characteristics of the cases of two NoV outbreaks in Changsha, China. (DOC 44 kb)

Additional file 4: Supplementary methods. (DOC 299 kb)

Additional file 5: Figure S1. Parameters estimation. (TIF 286 kb)

Additional file 6: Supplementary result. (DOC 25 kb)

Additional file 7: Table S4. Simulation of the best execution time of different interventions and the best period of school closure in two outbreaks in Changsha, 2014. (DOC 72 kb)

Additional file 8: Supplementary material. (DOC 791 kb)

Additional file 9: Table S5. Grade and class information of the cases in the second outbreak from December 18 to 24. (DOC 49 kb)

## Abbreviations

DO: Duration of outbreak
NoV: Norovirus
ODE: Ordinary differential equation
SEIARW: Susceptible-Exposed-Infectious/asymptomatic-Removed-Water
TARs: Total attack rates
