# Supplementary material for: Evidence-Based interventions of Norovirus outbreaks in China
Source: BMC Public Health. 2016 Oct 12;16:1072. doi: 10.1186/s12889-016-3716-3 (PMC5059926; doi:10.1186/s12889-016-3716-3)
Supplement: Supplementary file 1 — Summary of some of the reported waterborne NoV outbreaks since 2000. (DOC 97 kb) [file 12889_2016_3716_MOESM1_ESM.doc]

**Supplementary t**able 1 Summary of some of the reported waterborne NoV outbreaks since 2000

| Time | Location | Number of affected | Number of infected | DIR^&^ | TAR | DO* | Identified/predicted source of infection | Reference |
| --- | --- | --- | --- | --- | --- | --- | --- | --- |
| September 2009 | a small town school in semi-tropical Guangdong province, southern China | 5769 | 108 | 4 | 1.87 | 8 | Well water for household purposes | 1 |
| February 27-March 13, 2011 | Santo Stefano di Quisquina, Agrigento, Sicily, Italy | 4,965 | 156 | 5 | 3.14 | 15 | A well and springs supplying the public water network | 2 |
| March 2008 | Hangzhou city, Zhejiang province, China | 7694 | 336 | NA | 4.37 | NA | Bottled water | 3 |
| July 2006 | a popular ski resort in southern New Zealand | Up to 4280 | 218 | 6 | About 5.09 | 10 | resort drinking water | 4 |
| early spring 2012 | a hotel or neighbouring resort in southern New Zealand | Up to 320 | 53 | 10 | 16.56 | 20 | drinking water and the wider environment | 5 |
| September 2008 | Lilla Edet, Sweden | 13,000 | About 2,400 | 7 | About18.46 | 23 | municipal drinking water | 6 |
| December 6-13, 2010 | Hemiksem, Belgium | 1185 persons living in the 528 responding households | 222 persons | 8 | 18.73 | 28 | Drinking tap water from river water | 7 |
| January 31-February 1, 2004 | a swimming club in Vermont, USA | 189 | 53 | 4 | 28.04 | NA | Swimming pool | 8 |
| May 2004 | two hotels 300 m apart, on Jeju Island, South Korea | 516 | 194 | NA | 37.60 | NA | groundwater | 9 |
| The first week after the school summer holidays | a school in Borges Blanques (Lleida, Spain) | 213 | 96 | NA | 45.07 | NA | school drinking water | 10 |
| December 12-13, 2012 | a geographical distinct area in Denmark | 256 | 130 | 1 | 50.78 | 9 | drinking tap-water | 11 |
| 2007 | the town of Nokia in Southern Finland | 323 | 174 | NA | 53.87 | NA | drinking water | 12 |
| Easter 2009 | a small Swedish village | 270 | 173 | 8 | 64.07 | 11 | A well supplying the public water network | 13 |
| October 2001 | Wyoming, the US | 111 | 84 | NA | 75.68 | 30 | Drinking water from a saloon’s well | 14 |
| February 25-March 5, 2009 | A resort in Guatemala | 119 | 92 | 5 | 77.31 | 11 | resort drinking water | 15 |
| July 2007 | South Limburg, the Netherlands | 84 | 71 | NA | 84.52 | 13 | drinking water from a farmer's well | 16 |
| December 18-27, 2014 | Changsha city, Hunan province, China | 1553 | 30 | 6 | 2.14 | 23 | unknown | This study |
| November 28-December 16, 2014 | Changsha city, Hunan province, China | 643 | 159 | 9 | 19.78 | 23 | Water pipe | This study |

&: duration from symptom onset of index case to the outbreak was reported

*: duration between the dates of index and last case

#: representing the data of some cases only
