# Supplementary material for: Evidence-Based interventions of Norovirus outbreaks in China
Source: BMC Public Health. 2016 Oct 12;16:1072. doi: 10.1186/s12889-016-3716-3 (PMC5059926; doi:10.1186/s12889-016-3716-3)
Supplement: Supplementary file 2 — Parameter definitions and values for sensitivity analysis. (DOC 89 kb) [file 12889_2016_3716_MOESM2_ESM.doc]

**Supplementary t**able 2. Parameter definitions and values for sensitivity analysis

| Parameter | Description | Unit | Range | Value | Reference |
| --- | --- | --- | --- | --- | --- |
| *ω* | Relative incubation rate* | day^-1^ | 0.5~2 | 1 | 17,18 |
| *ω′* | Relative latency rate* | day^-1^ | 0.2~2 | 1 | 18 |
| *p* | Proportion of the asymptomatic | 1 | 0~0.5 | 0.3 | 19–21 |
| *γ* | Recovery rate of the infected | day^-1^ | 0.1667~1 | 0.3333 | 22–26 |
| *γ'* | Recovery rate of the asymptomatic | day^-1^ | 0.01852-0.0909 | 0.03846 | 19 |
| *ε* | Relative norovirus survival rate | day^-1^ | 0.03571-0.1429 | 0.1 | 27–34 |

*: Incubation period is the time elapsed between infected and symptoms are first apparent, and latent period means the time from infected to infectiousness, in this table, incubation period =1/*ω*, latent period =1/*ω′*.
