# Supplementary material for: Evidence-Based interventions of Norovirus outbreaks in China
Source: BMC Public Health. 2016 Oct 12;16:1072. doi: 10.1186/s12889-016-3716-3 (PMC5059926; doi:10.1186/s12889-016-3716-3)
Supplement: Supplementary file 3 — Demographic and clinical characteristics of the cases of two NoV outbreaks in Changsha, China. (DOC 44 kb) [file 12889_2016_3716_MOESM3_ESM.doc]

**Supplementary table 3.** Demographic and clinical characteristics of the cases of two NoV outbreaks in Changsha, China

| Variables | outbreak 1  (n=159) | outbreak 2  (n=30) |
| --- | --- | --- |
| Sex (No. of subjects [%]) |  |  |
| Male | 73 (45.91) | 19 (63.33) |
| Female | 86 (54.09) | 11 ( 36.67) |
| Age (No. of subjects [%]) |  |  |
| 0- | 10 (6.29) | 0 (0.00) |
| 15- | 5 (3.14) | 30 (100.00) |
| 25- | 4 (2.52) | 0 (0.00) |
| 35- | 19 (11.95) | 0 (0.00) |
| 45- | 35 (22.01) | 0 (0.00) |
| 55- | 37 (23.27) | 0 (0.00) |
| 65- | 44 (27.67) | 0 (0.00) |
| 75- | 5 (3.14) | 0 (0.00) |
| Clinical symptoms (No. of subjects [%]) |  |  |
| Diarrhea | 99 (62.26) | 19 (63.33) |
| Nausea | 82 (51.57) | 21 (70.00) |
| Abdominal pain | 79 (49.69) | 20 (66.67) |
| Abdominal distension | 72 (45.28) | 0 (0.00) |
| Vomit | 74 (46.54) | 27 (90.00) |
| Chill | 29 (18.24) | 0 (0.00) |
| Fever | 0 (0.00) | 4 (13.33) |
| Headache | 0 (0.00) | 7 (23.33) |
| Dizziness | 0 (0.00) | 15 (50.00) |
