# Supplementary figures and images for: Evidence-Based interventions of Norovirus outbreaks in China
Source: BMC Public Health. 2016 Oct 12;16:1072. doi: 10.1186/s12889-016-3716-3 (PMC5059926; doi:10.1186/s12889-016-3716-3)

**Supplementary Figure 1.** Parameters estimation

**
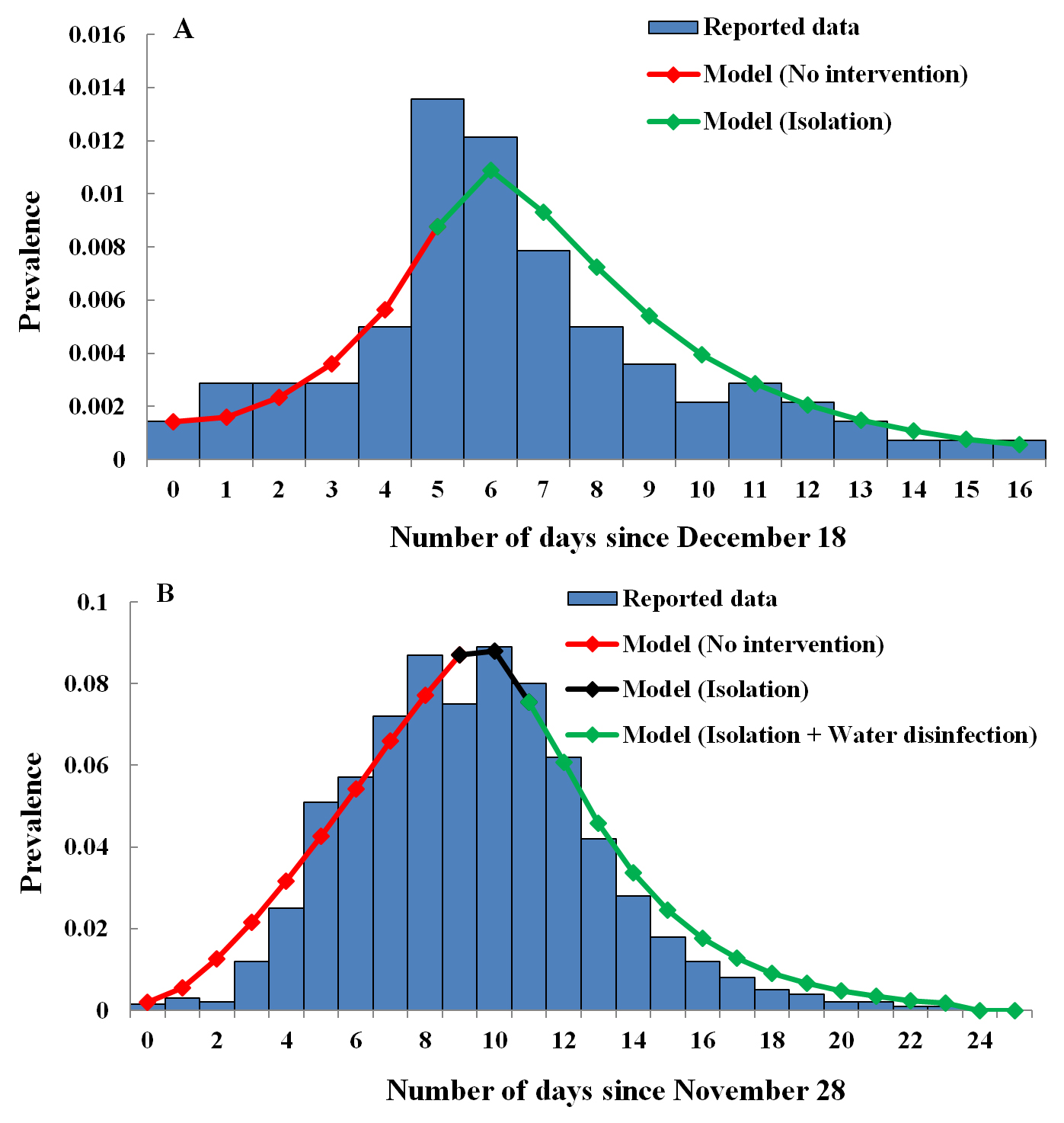
**

Supplement: Supplementary file 5 — Parameters estimation. (TIF 286 kb) [file 12889_2016_3716_MOESM5_ESM.doc]
