# Supplementary material for: Evidence-Based interventions of Norovirus outbreaks in China
Source: BMC Public Health. 2016 Oct 12;16:1072. doi: 10.1186/s12889-016-3716-3 (PMC5059926; doi:10.1186/s12889-016-3716-3)
Supplement: Supplementary file 6 — Supplementary result. (DOC 25 kb) [file 12889_2016_3716_MOESM6_ESM.doc]

**Supplementary result**

A water distribution network and storage devices had been used by the neighborhood for more than 40 years but storage wells and water towers had not been decontaminated and disinfected for many years. Water service pipes and sewer pipes were separated within a distance of 5 feet. Potable water service pipes were above a cesspool. The residents reflected that the yellow or green turbid water contains coarse suspended particles and the smell of mud. They denied the habit of drinking unboiled water, yet using it to clean fruit, cutlery and food. They had neither recent history of feast, nor dinning together with a lot of people. Their eating habits and food choice remained the same. Most of them ate homegrown vegetables and the meat was mainly acquired from local markets.
