# Supplementary material for: Evidence-Based interventions of Norovirus outbreaks in China
Source: BMC Public Health. 2016 Oct 12;16:1072. doi: 10.1186/s12889-016-3716-3 (PMC5059926; doi:10.1186/s12889-016-3716-3)
Supplement: Supplementary file 7 — Simulation of the best execution time of different interventions and the best period of school closure in two outbreaks in Changsha, 2014. (DOC 72 kb) [file 12889_2016_3716_MOESM7_ESM.doc]

Supplementary Table 4. Simulation of the best execution time of different interventions and the best period of school closure in two outbreaks in Changsha, 2014

| Intervention | TAR^a^ (%) | DO^b^ (day) | Peak day | Number of peak cases |
| --- | --- | --- | --- | --- |
| Outbreak 1 |  |  |  |  |
| Iso (begin on day 3) | 70.00 | 207 | Day 6 | 19 |
| Iso (begin on day 5) | 70.00 | 204 | Day 6 | 32 |
| Iso (begin on day 7) | 70.00 | 197 | Day 8 | 45 |
| Iso (begin on day 9) | 70.00 | 189 | Day 10 | 57 |
| Iso (begin on day 11) | 70.00 | 178 | Day 11 | 65 |
| Iso (begin on day 13) | 70.00 | 166 | Day 13 | 67 |
| Iso (begin on day 15) | 70.00 | 155 | Day 13 | 67 |
| W_dis_ (begin on day 3) | 51.18 | 48 | Day 15 | 45 |
| W_dis_ (begin on day 5) | 52.17 | 45 | Day 13 | 51 |
| W_dis_ (begin on day 7) | 53.07 | 43 | Day 12 | 56 |
| W_dis_ (begin on day 9) | 53.89 | 42 | Day11 | 62 |
| W_dis_ (begin on day 11) | 54.64 | 41 | Day 12 | 66 |
| W_dis_ (begin on day 13) | 55.34 | 40 | Day 13 | 67 |
| W_dis_ (begin on day 15) | 55.99 | 40 | Day 13 | 67 |
| Iso + W_dis_ (begin on day 3) | 5.06 | 13 | Day 4 | 17 |
| Iso + W_dis_ (begin on day 5) | 10.33 | 17 | Day 6 | 30 |
| Iso + W_dis_ (begin on day 7) | 17.17 | 20 | Day 8 | 44 |
| Iso + W_dis_ (begin on day 9) | 24.93 | 22 | Day 9 | 56 |
| Iso + W_dis_ (begin on day 11) | 32.64 | 25 | Day 11 | 65 |
| Iso + W_dis_ (begin on day 13) | 39.45 | 27 | Day 13 | 67 |
| Iso + W_dis_ (begin on day 15) | 44.92 | 28 | Day 13 | 67 |
| None | 70.00 | 450 | Day 13 | 67 |
| Outbreak 2. |  |  |  |  |
| Iso (begin on day 3) | 0.87 | 8 | Day 4 | 6 |
| Iso (begin on day 5) | 2.26 | 13 | Day 6 | 15 |
| Iso (begin on day 7) | 5.50 | 17 | Day 8 | 36 |
| Iso (begin on day 9) | 12.36 | 21 | Day 10 | 78 |
| Iso (begin on day 11) | 24.41 | 25 | Day 12 | 146 |
| Iso (begin on day 13) | 39.75 | 28 | Day 14 | 215 |
| Iso (begin on day 15) | 52.67 | 30 | Day 15 | 252 |
| Sc (7 days) | 67.23 | 50 | Day 26 | 233 |
| Sc (8 days) | 67.22 | 52 | Day 28 | 233 |
| Sc (9 days) | 67.21 | 54 | Day 30 | 231 |
| Sc (10 days) | 2.26 | 13 | Day 6 | 15 |
| Sc (11 days) | 2.26 | 13 | Day 6 | 15 |
| Sc (12 days) | 2.26 | 13 | Day 6 | 15 |
| Sc (13 days) | 2.26 | 13 | Day 6 | 15 |
| Iso (begin on day 3) + Sc (7 days) | 0.87 | 8 | Day 4 | 6 |
| Iso (begin on day 5) + Sc (8 days) | 2.26 | 13 | Day 6 | 15 |
| Iso (begin on day 7) + Sc (9 days) | 2.26 | 13 | Day 6 | 15 |
| Iso (begin on day 9) + Sc (10 days) | 2.26 | 13 | Day 6 | 15 |
| Iso (begin on day 11) + Sc (11 days) | 2.26 | 13 | Day 6 | 15 |
| Iso (begin on day 13) + Sc (12 days) | 2.26 | 13 | Day 6 | 15 |
| Iso (begin on day 15) + Sc (13 days) | 2.26 | 13 | Day 6 | 15 |
| None | 67.45 | 944 |  |  |

^a^, Total attack rate; ^b^, duration of outbreak; Iso, Isolation; W_dis_, Water disinfection; Sc, School closure begin at the reported day.
