# Supplementary material for: Evidence-Based interventions of Norovirus outbreaks in China
Source: BMC Public Health. 2016 Oct 12;16:1072. doi: 10.1186/s12889-016-3716-3 (PMC5059926; doi:10.1186/s12889-016-3716-3)
Supplement: Supplementary file 8 — Supplementary material. (DOC 791 kb) [file 12889_2016_3716_MOESM8_ESM.doc]

**Supplementary Figure 1.** Parameters estimation

**
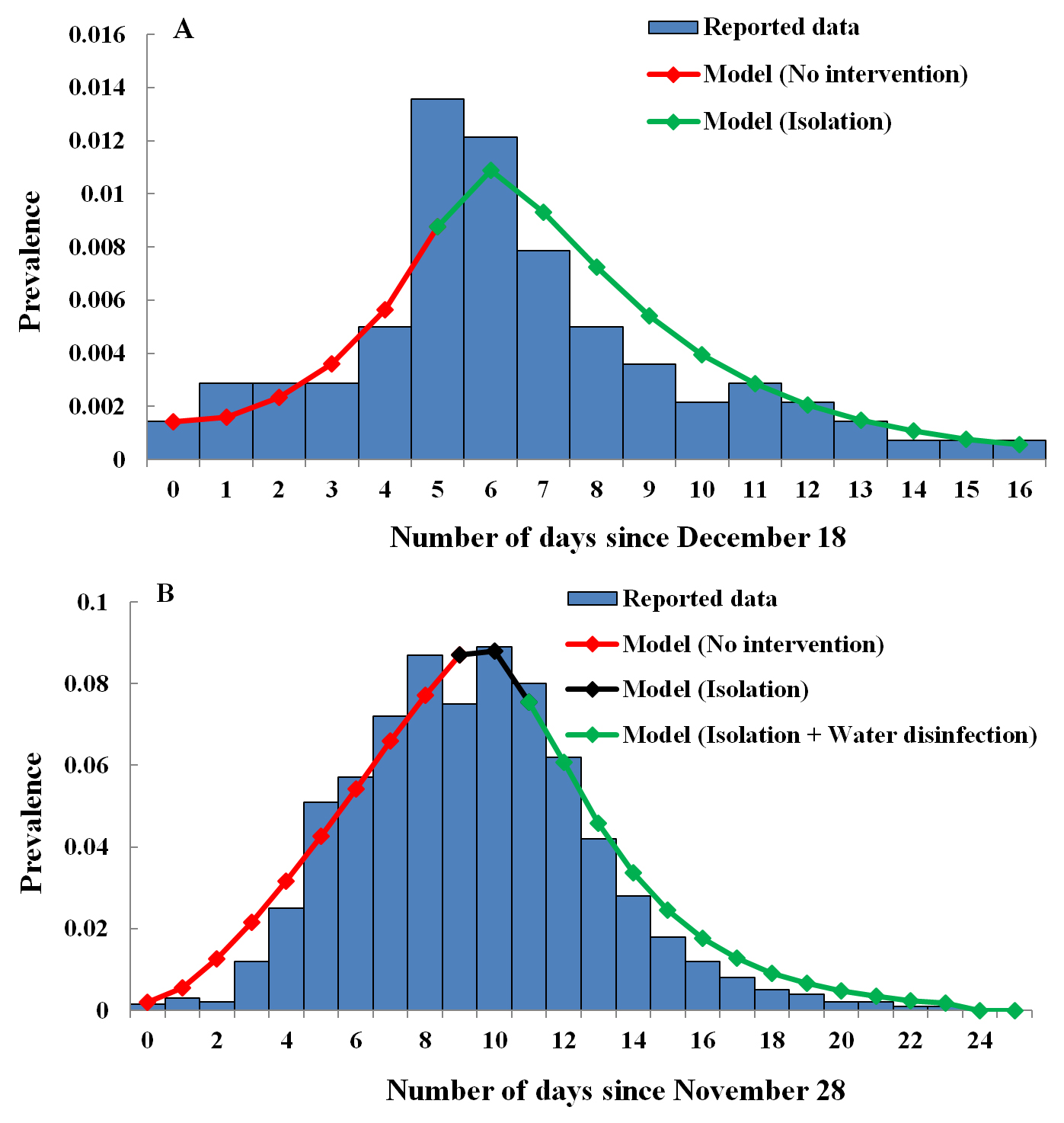
**

**Supplementary result**

A water distribution network and storage devices had been used by the neighborhood for more than 40 years but storage wells and water towers had not been decontaminated and disinfected for many years. Water service pipes and sewer pipes were separated within a distance of 5 feet. Potable water service pipes were above a cesspool. The residents reflected that the yellow or green turbid water contains coarse suspended particles and the smell of mud. They denied the habit of drinking unboiled water, yet using it to clean fruit, cutlery and food. They had neither recent history of feast, nor dinning together with a lot of people. Their eating habits and food choice remained the same. Most of them ate homegrown vegetables and the meat was mainly acquired from local markets.

**Supplementary t**able 1 Summary of some of the reported waterborne NoV outbreaks since 2000

| Time | Location | Number of affected | Number of infected | DIR^&^ | TAR | DO* | Identified/predicted source of infection | Reference |
| --- | --- | --- | --- | --- | --- | --- | --- | --- |
| September 2009 | a small town school in semi-tropical Guangdong province, southern China | 5769 | 108 | 4 | 1.87 | 8 | Well water for household purposes | 1 |
| February 27-March 13, 2011 | Santo Stefano di Quisquina, Agrigento, Sicily, Italy | 4,965 | 156 | 5 | 3.14 | 15 | A well and springs supplying the public water network | 2 |
| March 2008 | Hangzhou city, Zhejiang province, China | 7694 | 336 | NA | 4.37 | NA | Bottled water | 3 |
| July 2006 | a popular ski resort in southern New Zealand | Up to 4280 | 218 | 6 | About 5.09 | 10 | resort drinking water | 4 |
| early spring 2012 | a hotel or neighbouring resort in southern New Zealand | Up to 320 | 53 | 10 | 16.56 | 20 | drinking water and the wider environment | 5 |
| September 2008 | Lilla Edet, Sweden | 13,000 | About 2,400 | 7 | About18.46 | 23 | municipal drinking water | 6 |
| December 6-13, 2010 | Hemiksem, Belgium | 1185 persons living in the 528 responding households | 222 persons | 8 | 18.73 | 28 | Drinking tap water from river water | 7 |
| January 31-February 1, 2004 | a swimming club in Vermont, USA | 189 | 53 | 4 | 28.04 | NA | Swimming pool | 8 |
| May 2004 | two hotels 300 m apart, on Jeju Island, South Korea | 516 | 194 | NA | 37.60 | NA | groundwater | 9 |
| The first week after the school summer holidays | a school in Borges Blanques (Lleida, Spain) | 213 | 96 | NA | 45.07 | NA | school drinking water | 10 |
| December 12-13, 2012 | a geographical distinct area in Denmark | 256 | 130 | 1 | 50.78 | 9 | drinking tap-water | 11 |
| 2007 | the town of Nokia in Southern Finland | 323 | 174 | NA | 53.87 | NA | drinking water | 12 |
| Easter 2009 | a small Swedish village | 270 | 173 | 8 | 64.07 | 11 | A well supplying the public water network | 13 |
| October 2001 | Wyoming, the US | 111 | 84 | NA | 75.68 | 30 | Drinking water from a saloon’s well | 14 |
| February 25-March 5, 2009 | A resort in Guatemala | 119 | 92 | 5 | 77.31 | 11 | resort drinking water | 15 |
| July 2007 | South Limburg, the Netherlands | 84 | 71 | NA | 84.52 | 13 | drinking water from a farmer's well | 16 |
| December 18-27, 2014 | Changsha city, Hunan province, China | 1553 | 30 | 6 | 2.14 | 23 | unknown | This study |
| November 28-December 16, 2014 | Changsha city, Hunan province, China | 643 | 159 | 9 | 19.78 | 23 | Water pipe | This study |

&: duration from symptom onset of index case to the outbreak was reported

*: duration between the dates of index and last case

#: representing the data of some cases only

**Supplementary t**able 2. Parameter definitions and values for sensitivity analysis

| Parameter | Description | Unit | Range | Value | Reference |
| --- | --- | --- | --- | --- | --- |
| *ω* | Relative incubation rate* | day^-1^ | 0.5~2 | 1 | 17,18 |
| *ω′* | Relative latency rate* | day^-1^ | 0.2~2 | 1 | 18 |
| *p* | Proportion of the asymptomatic | 1 | 0~0.5 | 0.3 | 19–21 |
| *γ* | Recovery rate of the infected | day^-1^ | 0.1667~1 | 0.3333 | 22–26 |
| *γ'* | Recovery rate of the asymptomatic | day^-1^ | 0.01852-0.0909 | 0.03846 | 19 |
| *ε* | Relative norovirus survival rate | day^-1^ | 0.03571-0.1429 | 0.1 | 27–34 |

*: Incubation period is the time elapsed between infected and symptoms are first apparent, and latent period means the time from infected to infectiousness, in this table, incubation period =1/*ω*, latent period =1/*ω′*.

**Supplementary table 3.** Demographic and clinical characteristics of the cases of two NoV outbreaks in Changsha, China

| Variables | outbreak 1  (n=159) | outbreak 2  (n=30) |
| --- | --- | --- |
| Sex (No. of subjects [%]) |  |  |
| Male | 73 (45.91) | 19 (63.33) |
| Female | 86 (54.09) | 11 ( 36.67) |
| Age (No. of subjects [%]) |  |  |
| 0- | 10 (6.29) | 0 (0.00) |
| 15- | 5 (3.14) | 30 (100.00) |
| 25- | 4 (2.52) | 0 (0.00) |
| 35- | 19 (11.95) | 0 (0.00) |
| 45- | 35 (22.01) | 0 (0.00) |
| 55- | 37 (23.27) | 0 (0.00) |
| 65- | 44 (27.67) | 0 (0.00) |
| 75- | 5 (3.14) | 0 (0.00) |
| Clinical symptoms (No. of subjects [%]) |  |  |
| Diarrhea | 99 (62.26) | 19 (63.33) |
| Nausea | 82 (51.57) | 21 (70.00) |
| Abdominal pain | 79 (49.69) | 20 (66.67) |
| Abdominal distension | 72 (45.28) | 0 (0.00) |
| Vomit | 74 (46.54) | 27 (90.00) |
| Chill | 29 (18.24) | 0 (0.00) |
| Fever | 0 (0.00) | 4 (13.33) |
| Headache | 0 (0.00) | 7 (23.33) |
| Dizziness | 0 (0.00) | 15 (50.00) |

Supplementary Table 4. Simulation of the best execution time of different interventions and the best period of school closure in two outbreaks in Changsha, 2014

| Intervention | TAR^a^ (%) | DO^b^ (day) | Peak day | Number of peak cases |
| --- | --- | --- | --- | --- |
| Outbreak 1 |  |  |  |  |
| Iso (begin on day 3) | 70.00 | 207 | Day 6 | 19 |
| Iso (begin on day 5) | 70.00 | 204 | Day 6 | 32 |
| Iso (begin on day 7) | 70.00 | 197 | Day 8 | 45 |
| Iso (begin on day 9) | 70.00 | 189 | Day 10 | 57 |
| Iso (begin on day 11) | 70.00 | 178 | Day 11 | 65 |
| Iso (begin on day 13) | 70.00 | 166 | Day 13 | 67 |
| Iso (begin on day 15) | 70.00 | 155 | Day 13 | 67 |
| W_dis_ (begin on day 3) | 51.18 | 48 | Day 15 | 45 |
| W_dis_ (begin on day 5) | 52.17 | 45 | Day 13 | 51 |
| W_dis_ (begin on day 7) | 53.07 | 43 | Day 12 | 56 |
| W_dis_ (begin on day 9) | 53.89 | 42 | Day11 | 62 |
| W_dis_ (begin on day 11) | 54.64 | 41 | Day 12 | 66 |
| W_dis_ (begin on day 13) | 55.34 | 40 | Day 13 | 67 |
| W_dis_ (begin on day 15) | 55.99 | 40 | Day 13 | 67 |
| Iso + W_dis_ (begin on day 3) | 5.06 | 13 | Day 4 | 17 |
| Iso + W_dis_ (begin on day 5) | 10.33 | 17 | Day 6 | 30 |
| Iso + W_dis_ (begin on day 7) | 17.17 | 20 | Day 8 | 44 |
| Iso + W_dis_ (begin on day 9) | 24.93 | 22 | Day 9 | 56 |
| Iso + W_dis_ (begin on day 11) | 32.64 | 25 | Day 11 | 65 |
| Iso + W_dis_ (begin on day 13) | 39.45 | 27 | Day 13 | 67 |
| Iso + W_dis_ (begin on day 15) | 44.92 | 28 | Day 13 | 67 |
| None | 70.00 | 450 | Day 13 | 67 |
| Outbreak 2. |  |  |  |  |
| Iso (begin on day 3) | 0.87 | 8 | Day 4 | 6 |
| Iso (begin on day 5) | 2.26 | 13 | Day 6 | 15 |
| Iso (begin on day 7) | 5.50 | 17 | Day 8 | 36 |
| Iso (begin on day 9) | 12.36 | 21 | Day 10 | 78 |
| Iso (begin on day 11) | 24.41 | 25 | Day 12 | 146 |
| Iso (begin on day 13) | 39.75 | 28 | Day 14 | 215 |
| Iso (begin on day 15) | 52.67 | 30 | Day 15 | 252 |
| Sc (7 days) | 67.23 | 50 | Day 26 | 233 |
| Sc (8 days) | 67.22 | 52 | Day 28 | 233 |
| Sc (9 days) | 67.21 | 54 | Day 30 | 231 |
| Sc (10 days) | 2.26 | 13 | Day 6 | 15 |
| Sc (11 days) | 2.26 | 13 | Day 6 | 15 |
| Sc (12 days) | 2.26 | 13 | Day 6 | 15 |
| Sc (13 days) | 2.26 | 13 | Day 6 | 15 |
| Iso (begin on day 3) + Sc (7 days) | 0.87 | 8 | Day 4 | 6 |
| Iso (begin on day 5) + Sc (8 days) | 2.26 | 13 | Day 6 | 15 |
| Iso (begin on day 7) + Sc (9 days) | 2.26 | 13 | Day 6 | 15 |
| Iso (begin on day 9) + Sc (10 days) | 2.26 | 13 | Day 6 | 15 |
| Iso (begin on day 11) + Sc (11 days) | 2.26 | 13 | Day 6 | 15 |
| Iso (begin on day 13) + Sc (12 days) | 2.26 | 13 | Day 6 | 15 |
| Iso (begin on day 15) + Sc (13 days) | 2.26 | 13 | Day 6 | 15 |
| None | 67.45 | 944 |  |  |

^a^, Total attack rate; ^b^, duration of outbreak; Iso, Isolation; W_dis_, Water disinfection; Sc, School closure begin at the reported day.

Supplementary Table 5. **Grade and class information of the cases in the second outbreak from December 18 to 24**

| ID | Grade | Class | Month |
| --- | --- | --- | --- |
| 5 | 1 | 276 | 12 |
| 20 | 1 | 276 | 12 |
| 7 | 1 | 277 | 12 |
| 21 | 1 | 277 | 12 |
| 22 | 1 | 277 | 12 |
| 23 | 1 | 277 | 12 |
| 29 | 1 | 278 | 12 |
| 2 | 1 | 280 | 12 |
| 6 | 1 | 280 | 12 |
| 12 | 1 | 280 | 12 |
| 24 | 1 | 280 | 12 |
| 25 | 1 | 280 | 12 |
| 26 | 1 | 280 | 12 |
| 30 | 1 | 280 | 12 |
| 1 | 3 | 263 | 12 |
| 13 | 3 | 263 | 12 |
| 27 | 3 | 266 | 12 |
| 3 | 2 | 269 | 12 |
| 8 | 2 | 269 | 12 |
| 4 | 2 | 270 | 12 |
| 9 | 2 | 270 | 12 |
| 10 | 2 | 270 | 12 |
| 14 | 2 | 270 | 12 |
| 15 | 2 | 272 | 12 |
| 16 | 2 | 272 | 12 |
| 17 | 2 | 273 | 12 |
| 11 | 2 | 274 | 12 |
| 18 | 2 | 274 | 12 |
| 19 | 2 | 274 | 12 |
| 28 | 2 | 274 | 12 |

**Supplementary methods**

**No intervention**

According to the natural history of norovirus infection, we built a Susceptible–Exposed–Infectious/asymptomatic–Removed–Water (SEIARW) model, where individuals are characterized according to their epidemiological status as susceptible (*S*), exposed (*E*, infected but not yet fully contagious), infectious (*I*), asymptomatic (*A*), and recovered (*R*); *W* denotes the reservoir (water) compartment. A deterministic model was developed on the basis of the following facts and assumptions:

(1) Transmission occurs via either a person–person or a person–water–person route.

(2) No fatality was identified in the two outbreaks investigated. Thus, fatal cases were not included in the model.

(3) Infection during an outbreak confers permanent immunity.

(4) The transmission of norovirus during an outbreak occurs within a closed system, defined as a system with no migration in or out; adjustment for births and natural deaths was not included in the model.


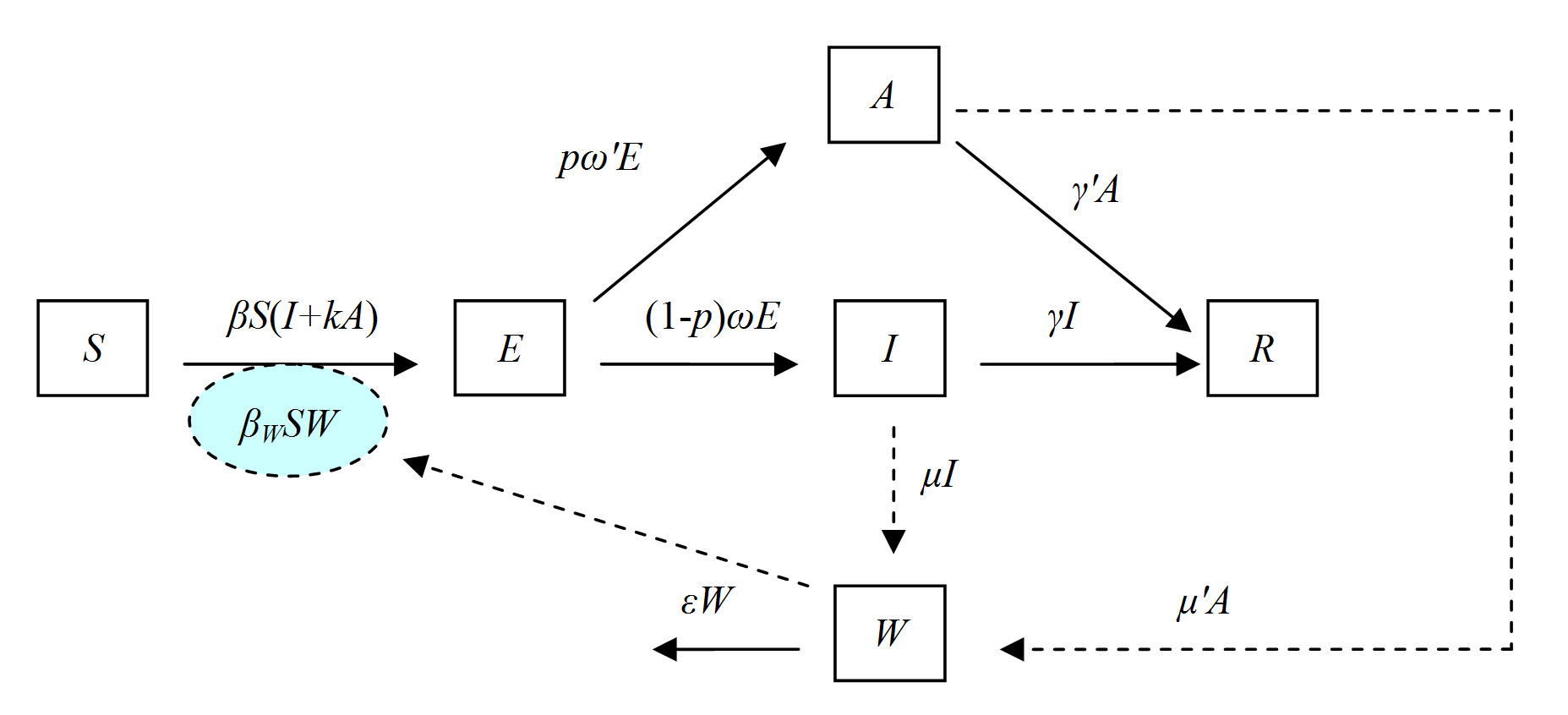


**Figure 1. Flowchart of development of the SEIARW model**

Figure 1 depicts the SEIARW model. Susceptible individuals become infected (i.e., move from *S* to *E*) by contact with either infected/asymptomatic individuals or contaminated water at rates of *βSI*, *βkSA* and *β_W_SW* respectively, where *β* and *β_W_* are the probability of transmission per contact, *k* is the relative transmissibility of asymptomatic to symptomatic individuals. As exposed individuals become infectious after an incubation period, they move from *E* to *I* at a rate of (1-*p*)*ωE* and *E* to *A* at a rate of *pω'E*, where 1/*ω* is the incubation period, 1/*ω'* is the latent period of the disease and *p* is the proportion of asymptomatic individuals. After the infectious period has passed, infectious and asymptomatic individuals may move to *R* at a rate of *γI* and *γ'A* respectively, where 1/*γ* and 1/*γ'* are the infectious period of the *I* and *A*. Infectious and asymptomatic individuals can in turn contaminate the water compartment by shedding the pathogen into *W* at shedding rates of *μI* and *μ'A*, where *μ* and *μ'* are the shedding coefficients. The pathogen in *W* can subsequently leave the water compartment at a rate of *εW*, where 1/*ε* is the lifetime of the pathogen. The corresponding model equations are as follows:

$\left\{\begin{matrix} dS/dt=-\beta S(I+kA)-\beta_{W}SW \\ dE/dt=\beta S(I+kA)+\beta_{W}SW-(1-p)\omega E-p\omega'E \\ dI/dt=(1-p)\omega E-\gamma I \\ dA/dt=p\omega'E-\gamma'A \\ dR/dt=\gamma I+\gamma'A \\ dW/dt=\mu I+\mu'A-\varepsilon W \end{matrix}\right.$ (1)

It would be instructive to consider a rescaling of model (1) using dimensionless variables. If *N* is assumed to denote the total population size and *s* = *S/N*, *e* = *E/N*, *i* = *I/N*, *a* = *I/A*, *r*= *R/N*, *w* = *εW/μN*, *μ'=cμ*, *b* = *βN*, and *b_W_* = *μβ_W_N/ε*, the following rescaled model can be developed:

$\left\{\begin{matrix} ds/dt=-bs(i+ka)-b_{W}sw \\ de/dt=bs(i+ka)+b_{W}sw-(1-p)\omega e-p\omega'e \\ di/dt=(1-p)\omega e-\gamma i \\ da/dt=p\omega'e-\gamma'a \\ dr/dt=\gamma i+\gamma'a \\ dw/dt=\varepsilon(i+ca-w) \end{matrix}\right.$ (2)

Since the ease of its transmission, with a very low infectious dose of 18 virions, the transmissibility of the contagious water will be “0 or 1” phenomenon. It means that, for one thing, the water will be infectious after a case shedding the virus into the water, for the other thing, the water consist its transmissibility after it is contaminated even though more virus are shed into the water by more cases. Therefore, *w*(*t*) is a constant although virus are shed into the water by *i* and *a* continuously. That a susceptible is infected or not is determined by the route of contacting the water effectively or not, and this infection reflect in the parameter *b_W_*.

**Case isolation**

In practice, isolation aimed at the *i* population and was implemented from the date when CDC received report. On the first isolation day, all symptomatic cases were isolated; after that, a new case would be isolated upon showing symptoms. Milder norovirus infection cases were requested to stay home. Dedicated staff paid visits to ensure adherence, hygiene, and proper disinfection. More severe cases were hospitalized and isolated. Both cases were discharged after being free of symptoms for two days. In the case isolation model, neither the reservoir-to-person nor the person–to-person routes are viable means of transmission. Nevertheless, individuals in compartment *s* could become infected via the reservoir–to-person and asymptomatic-susceptible routes, leading to development of the following rescaled model:

$\left\{\begin{matrix} ds/dt=-b_{W}sw-bksa \\ de/dt=b_{W}sw+bksa-(1-p)\omega e-p\omega'e \\ di/dt=(1-p)\omega e-\gamma i \\ da/dt=p\omega'e-\gamma'a \\ dr/dt=\gamma i+\gamma'a \\ dw/dt=\varepsilon(ca-w) \end{matrix}\right.$ (3)

In addition, the effective implementation of case isolation needs other supplementary measures, such as disinfection of the environment which was contaminated by isolated case, inspection of all healthy persons, and put the institution of absence of class in school into practice, and so on. Therefore, case isolation in model (3) was a package which included supplementary measures.

**Water disinfection**

The model of water disinfection was show as model (4). In this model, the “water-person” route was cut off, which means *b_W_*=0. Transmission occurs via daily contact, it assumes that transmission occurs solely via the person–person route. Therefore, the variables *w*, *ε*, and *b_W_* are removed from the water disinfection model. The corresponding model equation is thus:

$\left\{\begin{matrix} ds/dt=-bs(i+ka) \\ de/dt=bs(i+ka)-(1-p)\omega e-p\omega'e \\ di/dt=(1-p)\omega e-\gamma i \\ da/dt=p\omega'e-\gamma'a \\ dr/dt=\gamma i+\gamma'a \end{matrix}\right.$ (4)

**Case isolation + water disinfection**

The model of “case isolation + water disinfection” was show as model (5). In this model, “patient–susceptible” and “water–susceptible” routes were cut off, leaving “asymptomatic– susceptible” route.

$\left\{\begin{matrix} ds/dt=-bksa \\ de/dt=bksa-(1-p)\omega e-p\omega'e \\ di/dt=(1-p)\omega e-\gamma i \\ da/dt=p\omega'e-\gamma'a \\ dr/dt=\gamma i+\gamma'a \end{matrix}\right.$ (5)

**School closure**

During a school closure, all the people in a school return home. Person–to-person and reservoir–to-person contacts are severed, making both *b* and *b_W_* become zero in effect in the school closure model. But when interval of school closure is over, all the people, including symptomatic and asymptomatic individuals, return school and transmission mode is back to SEIARW model. The corresponding model equation is thus:

$\left\{\begin{matrix} ds/dt=-f\left(b\right)s(i+ka)-f\left(b_{W}\right)sw \\ de/dt=f\left(b\right)s(i+ka)+f\left(b_{W}\right)sw-(1-p)\omega e-p\omega'e \\ di/dt=(1-p)\omega e-\gamma i \\ da/dt=p\omega'e-\gamma'a \\ dr/dt=\gamma i+\gamma'a \\ dw/dt=\varepsilon(i+ca-w) \end{matrix}\right.$ (6)

The function of parameters *b* and *b_W_* along with time is as follow:

$\left\{\begin{matrix} f\left(b\right)=b, \left(t ＜\pi_{1}\right) \\ f\left(b\right)=0, \left(\pi_{1}\leq t\leq\pi_{2}\right) \\ f\left(b\right)=b, \left(t ＞\pi_{2}\right) \\ f\left(b_{W}\right)=b_{W}, \left(t ＜\pi_{1}\right) \\ f\left(b_{W}\right)=0, \left(\pi_{1}\leq t\leq\pi_{2}\right) \\ f\left(b_{W}\right)=b_{W}, \left(t ＞\pi_{2}\right) \end{matrix}\right.$ (7)

In this function, *π*_1_ is initial time of school closure, *π*_2_ is the end of school closure, and Δ*π*=*π*_1_-*π*_2_ is the interval of school closure. We simulated the school closure of 7 days, 8 days, 9 days or 10 days, to examine the effects, which means Δ*π* = 7, 8, 9, 10.

**Combined-intervention strategies**

For the school outbreak in which the transmission route is “person-person”, we simulated combined intervention -“case isolation + school closure” to examine its impact. For the community outbreak in which the transmission routes are “water-person” and “person-person”, we simulated combined intervention -“case isolation + water disinfection” to examine its impact.

**Estimation of parameters**

Of *b*, *b_W_*, *k*, *ω*, *ω'*, *p*, *γ, γ'*, *c*, and *ε*, the 10 parameters in the model (Supplementary Table 2), *b* and *b_W_* could be estimated by curve fitting of SEIARW model and reported data regarding the outbreak. The incubation period of NoV cases ranges from 12 h to 48 h ^17,18^, we set incubation period equal to 1 day (*ω*=1) in our study. When a person was infected, he or she will shed the virus 36 h later at average, ranging from 18 h to 110 h, and the shedding status can persist for up to 26 days (ranges from 11 days to 54 days) ^22,19^, thus we valued latent period and infectious period equal to 1 day (*ω'*=1) and 26 days (*γ'*=0.03846) respectively. Duration of illness of NoV infection is 1-5 days ^19^, however, more prolonged courses of illness lasting 4-6 days ^25,24^ can also occur although it can only happen in a few cases. According to the Kaplan Criteria ^26^ which is widely used for diagnosis in the USA, NoV infected patients have the mean (or median) duration of illness of 1-3 days, we set this duration equal to 3 days (*γ*=0.3333) in our models. Up to 30% ^19–21^ of NoV infections are asymptomatic, therefore, *p*=0.3. Furthermore, asymptomatic persons can shed virus, albeit at lower titers than symptomatic persons ^19–21^, the role of asymptomatic infection in transmission and outbreaks of NoV remains unclear ^35^. Yet we set *k* as the transmissibility ratio of asymptomatic-to-symptomatic individuals, and *c* as the asymptomatic individual viral shedding coefficient. Then this two parameters were estimated by fitting the SEIARW models to the reported data we collected. Although NoV can survive from 7-12 days in external environment ^27,29^ and can even persist for up to 21-28 days ^34,32,30,33,28,31^, we set the average life time of NoV equal to 10 days (*ε*=0.1) in our study.

**Simulation methods**

Berkeley Madonna 8.3.18 and Microsoft Office Excel 2003 software were employed for model simulation and figure development, respectively. The Runge-Kutta method of order 4 with the tolerance set at 0.001 was used to perform curve fitting of SEIARW model and the reported data. While the curve fit is in progress, Berkeley Madonna displays the root mean square (RMS) deviation between the data and best run so far.

**Transmissibility and strategy assessment indicators**

Basic reproduction number (*R*_0_) was enrolled to evaluate the transmissibility of NoV. Here we use a final size equation ^36^ to calculate the *R*_0_ of different models. The final size equation is applicable to closed populations only, where the infection leads either to immunity or death. In this situation, the number of susceptibles can only decrease and the final fraction of susceptibles, S(∞), can be used to estimate *R*_0_:

$R_{0}=\frac{\ln S\left(\infty\right)}{S\left(\infty\right)-1}$ (8)

This was first recognized by Kermack & McKendrick (1927) ^37^; for a detailed derivation and discussion, see Diekmann & Heesterbeek (2000) ^38^, Hethcote (2000) ^39^ and Brauer (2002) ^40^.

We estimated TAR and duration of outbreak (DO) to assess the efficacy of the strategies for controlling the outbreak, where

$$TAR=\frac{n}{N}\times100\%$$

*n* is accumulative cases, *N* is total population.

$$DO=t_{2}-t_{1}$$

*n* is onset date of index case, *N* is recover date of last case.

**Sensitivity analysis**

Since six parameters, *ω*, *ω'*, *p*, *γ*, *γ'* and *ε*, were estimated by references, there was some uncertainty about them which might impact the results of models we built. In our study, sensitivity was tested by varying the two parameters which were split into 1000 values ranging from minimum to maximum of the reported values in literature (Supplementary Table 2).

**References**

1. Zhou X, Li H, Sun L, et al. Epidemiological and molecular analysis of a waterborne outbreak of norovirus GII.4. *Epidemiol Infect* 2012; 140: 2282–9.

2. Giammanco GM, Di Bartolo I, Purpari G, et al. Investigation and control of a Norovirus outbreak of probable waterborne transmission through a municipal groundwater system. *J Water Health* 2014; 12: 452–64.

3. Miao F, Deng J, Xie L. [Epidemiological investigation of a gastroenteritis outbreak caused by waterborne norovirus GG I]. *Zhonghua Liu Xing Bing Xue Za Zhi* 2009; 30: 99–100.

4. Hewitt J, Bell D, Simmons GC, et al. Gastroenteritis outbreak caused by waterborne norovirus at a New Zealand ski resort. *Appl Environ Microbiol* 2007; 73: 7853–7.

5. Jack S, Bell D, Hewitt J. Norovirus contamination of a drinking water supply at a hotel resort. *N Z Med J* 2013; 126: 98–107.

6. Larsson C, Andersson Y, Allestam G, et al. Epidemiology and estimated costs of a large waterborne outbreak of norovirus infection in Sweden. *Epidemiol Infect* 2014; 142: 592–600.

7. Braeye T, DE Schrijver K, Wollants E, et al. A large community outbreak of gastroenteritis associated with consumption of drinking water contaminated by river water, Belgium, 2010. *Epidemiol Infect* 2014; 1–9.

8. Centers for Disease Control and Prevention. An outbreak of norovirus gastroenteritis at a swimming club--Vermont, 2004. *MMWR Morb Mortal Wkly Rep* 2004; 53: 793–5.

9. Kim S-H, Cheon D-S, Kim J-H, et al. Outbreaks of gastroenteritis that occurred during school excursions in Korea were associated with several waterborne strains of norovirus. *J Clin Microbiol* 2005; 43: 4836–9.

10. Godoy P, Nuín C, Alsedà M, et al. Waterborne outbreak of gastroenteritis caused by Norovirus transmitted through drinking water. *Rev clínica española* 2006; 206: 435–7.

11. Van Alphen LB, Dorléans F, Schultz AC, et al. The application of new molecular methods in the investigation of a waterborne outbreak of norovirus in Denmark, 2012. *PLoS One* 2014; 9: e105053.

12. Laine J, Lumio J, Toikkanen S, et al. The duration of gastrointestinal and joint symptoms after a large waterborne outbreak of gastroenteritis in Finland in 2007--a questionnaire-based 15-month follow-up study. *PLoS One* 2014; 9: e85457.

13. Riera-Montes M, Brus Sjölander K, Allestam G, et al. Waterborne norovirus outbreak in a municipal drinking-water supply in Sweden. *Epidemiol Infect* 2011; 139: 1928–35.

14. Parshionikar SU, Willian-True S, Fout GS, et al. Waterborne outbreak of gastroenteritis associated with a norovirus. *Appl Environ Microbiol* 2003; 69: 5263–8.

15. Arvelo W, Sosa SM, Juliao P, et al. Norovirus outbreak of probable waterborne transmission with high attack rate in a Guatemalan resort. *J Clin Virol* 2012; 55: 8–11.

16. Ter Waarbeek HLG, Dukers-Muijrers NHTM, Vennema H, et al. Waterborne gastroenteritis outbreak at a scouting camp caused by two norovirus genogroups: GI and GII. *J Clin Virol* 2010; 47: 268–72.

17. Caul EO. Small round structured viruses: airborne transmission and hospital control. *Lancet* 1994; 343: 1240–2.

18. Hutson AM, Atmar RL, Estes MK. Norovirus disease: changing epidemiology and host susceptibility factors. *Trends Microbiol* 2004; 12: 279–87.

19. Atmar RL, Opekun AR, Gilger MA, et al. Norwalk virus shedding after experimental human infection. *Emerg Infect Dis* 2008; 14: 1553–7.

20. Graham DY, Jiang X, Tanaka T, et al. Norwalk virus infection of volunteers: new insights based on improved assays. *J Infect Dis* 1994; 170: 34–43.

21. Phillips G, Lopman B, Tam CC, et al. Diagnosing norovirus-associated infectious intestinal disease using viral load. *BMC Infect Dis* 2009; 9: 63.

22. Atmar RL, Estes MK. The epidemiologic and clinical importance of norovirus infection. *Gastroenterol Clin North Am* 2006; 35: 275–90, viii.

23. Kaplan JE, Schonberger LB, Varano G, et al. An outbreak of acute nonbacterial gastroenteritis in a nursing home. Demonstration of person-to-person transmission by temporal clustering of cases. *Am J Epidemiol* 1982; 116: 940–8.

24. Lopman BA, Reacher MH, Vipond IB, et al. Clinical manifestation of norovirus gastroenteritis in health care settings. *Clin Infect Dis* 2004; 39: 318–24.

25. Rockx B, De Wit M, Vennema H, et al. Natural history of human calicivirus infection: a prospective cohort study. *Clin Infect Dis* 2002; 35: 246–53.

26. Kaplan JE, Feldman R, Campbell DS, et al. The frequency of a Norwalk-like pattern of illness in outbreaks of acute gastroenteritis. *Am J Public Health* 1982; 72: 1329–32.

27. Mattison K, Karthikeyan K, Abebe M, et al. Survival of calicivirus in foods and on surfaces: experiments with feline calicivirus as a surrogate for norovirus. *J Food Prot* 2007; 70: 500–3.

28. Cheesbrough JS, Green J, Gallimore CI, et al. Widespread environmental contamination with Norwalk-like viruses (NLV) detected in a prolonged hotel outbreak of gastroenteritis. *Epidemiol Infect* 2000; 125: 93–8.

29. D’Souza DH, Sair A, Williams K, et al. Persistence of caliciviruses on environmental surfaces and their transfer to food. *Int J Food Microbiol* 2006; 108: 84–91.

30. Gallimore CI, Taylor C, Gennery AR, et al. Environmental monitoring for gastroenteric viruses in a pediatric primary immunodeficiency unit. *J Clin Microbiol* 2006; 44: 395–9.

31. Green J, Wright PA, Gallimore CI, et al. The role of environmental contamination with small round structured viruses in a hospital outbreak investigated by reverse-transcriptase polymerase chain reaction assay. *J Hosp Infect* 1998; 39: 39–45.

32. Jones EL, Kramer A, Gaither M, et al. Role of fomite contamination during an outbreak of norovirus on houseboats. *Int J Environ Health Res* 2007; 17: 123–31.

33. Kuusi M, Nuorti JP, Maunula L, et al. A prolonged outbreak of Norwalk-like calicivirus (NLV) gastroenteritis in a rehabilitation centre due to environmental contamination. *Epidemiol Infect* 2002; 129: 133–8.

34. Wu HM, Fornek M, Schwab KJ, et al. A norovirus outbreak at a long-term-care facility: the role of environmental surface contamination. *Infect Control Hosp Epidemiol* 2005; 26: 802–10.

35. CDC. Updated norovirus outbreak management and disease prevention guidelines. *MMWR Recomm Rep* 2011; 60: 1–18.

36. Heffernan JM, Smith RJ, Wahl LM. Perspectives on the basic reproductive ratio. *J R Soc Interface* 2005; 2: 281–293.

37. Kermack WO, McKendrick AG. A Contribution to the Mathematical Theory of Epidemics. *Proc R Soc A Math Phys Eng Sci* 1927; 115: 700–721.

38. Diekmann, O; Heesterbeek J. *Mathematical Epidemiology of Infectious Diseases: Model Building, Analysis and Interpretation*. 1st ed. Wiley, 2000.

39. Hethcote HW. The Mathematics of Infectious Diseases. *SIAM Rev* 2006; 42: 599–653.

40. Brauer F. *Mathematical Approaches for Emerging and Reemerging Infectious Diseases*. 2002.

**Supplementary figure legends**

**Figure 1 Curve fitting of data from two outbreaks.** A, curve fitting of reported data in a school since December 18. B, curve fitting of reported data in a community since November 28

**Supplementary table legends**

**Table 1 Summary of some of the reported waterborne NoV outbreaks since 2000**

**Table 2 Parameter definitions and values for sensitivity analysis**

**Table 3 Demographic and clinical characteristics of the cases of two NoV outbreaks in Changsha, China**

**Table 4 Simulation of the best execution time of different interventions and the best period of school closure in two outbreaks in Changsha, 2014**

**Table 5 Grade and class information of the cases in the second outbreak from December 18 to 24**
