# Supplementary material for: Evidence-Based interventions of Norovirus outbreaks in China
Source: BMC Public Health. 2016 Oct 12;16:1072. doi: 10.1186/s12889-016-3716-3 (PMC5059926; doi:10.1186/s12889-016-3716-3)
Supplement: Supplementary file 9 — Grade and class information of the cases in the second outbreak from December 18 to 24. (DOC 49 kb) [file 12889_2016_3716_MOESM9_ESM.doc]

Supplementary Table 5. **Grade and class information of the cases in the second outbreak from December 18 to 24**

| ID | Grade | Class | Month |
| --- | --- | --- | --- |
| 5 | 1 | 276 | 12 |
| 20 | 1 | 276 | 12 |
| 7 | 1 | 277 | 12 |
| 21 | 1 | 277 | 12 |
| 22 | 1 | 277 | 12 |
| 23 | 1 | 277 | 12 |
| 29 | 1 | 278 | 12 |
| 2 | 1 | 280 | 12 |
| 6 | 1 | 280 | 12 |
| 12 | 1 | 280 | 12 |
| 24 | 1 | 280 | 12 |
| 25 | 1 | 280 | 12 |
| 26 | 1 | 280 | 12 |
| 30 | 1 | 280 | 12 |
| 1 | 3 | 263 | 12 |
| 13 | 3 | 263 | 12 |
| 27 | 3 | 266 | 12 |
| 3 | 2 | 269 | 12 |
| 8 | 2 | 269 | 12 |
| 4 | 2 | 270 | 12 |
| 9 | 2 | 270 | 12 |
| 10 | 2 | 270 | 12 |
| 14 | 2 | 270 | 12 |
| 15 | 2 | 272 | 12 |
| 16 | 2 | 272 | 12 |
| 17 | 2 | 273 | 12 |
| 11 | 2 | 274 | 12 |
| 18 | 2 | 274 | 12 |
| 19 | 2 | 274 | 12 |
| 28 | 2 | 274 | 12 |
